# Constitutive expression of AhR and *BRCA-1* promoter CpG hypermethylation as biomarkers of ERα-negative breast tumorigenesis

**DOI:** 10.1186/s12885-015-2044-9

**Published:** 2015-12-29

**Authors:** Donato F. Romagnolo, Andreas J. Papoutsis, Christina Laukaitis, Ornella I. Selmin

**Affiliations:** Department of Nutritional Sciences, The University of Arizona, 303 Shantz Bldg, Tucson, AZ 85721-0038 USA; The University of Arizona Cancer Center, 1515 N. Campbell Avenue, 3999A, Tucson, AZ 85724-5024 USA; Department of Medicine, University of Arizona College of Medicine, The University of Arizona, Tucson, AZ USA

**Keywords:** *BRCA-1*, AhR, CpG methylation, Epigenetics, SAhRMs, ERα, Breast cancer

## Abstract

**Background:**

Only 5–10 % of breast cancer cases is linked to germline mutations in the *BRCA-1* gene and occurs early in life. Conversely, sporadic breast tumors, which represent 90-95 % of breast malignancies, have lower BRCA-1 expression, but not mutated *BRCA-1* gene, and tend to occur later in life in combination with other genetic alterations and/or environmental exposures. The latter may include environmental and dietary factors that activate the aromatic hydrocarbon receptor (AhR). Therefore, understanding if changes in expression and/or activation of the AhR are associated with somatic inactivation of the *BRCA-1* gene may provide clues for breast cancer therapy.

**Methods:**

We evaluated *Brca-1* CpG promoter methylation and expression in mammary tumors induced in Sprague–Dawley rats with the AhR agonist and mammary carcinogen 7,12-dimethyl-benzo(a)anthracene (DMBA). Also, we tested in human estrogen receptor (ER)α-negative sporadic UACC-3199 and ERα-positive MCF-7 breast cancer cells carrying respectively, hyper- and hypomethylated *BRCA-1* gene, if the treatment with the AhR antagonist α-naphthoflavone (αNF) modulated BRCA-1 and ERα expression. Finally, we examined the association between expression of *AhR* and *BRCA-1* promoter CpG methylation in human triple-negative (TNBC), luminal-A (LUM-A), LUM-B, and epidermal growth factor receptor-2 (HER-2)-positive breast tumor samples.

**Results:**

Mammary tumors induced with DMBA had reduced BRCA-1 and ERα expression; higher *Brca-1* promoter CpG methylation; increased expression of *Ahr* and its downstream target *Cyp1b1*; and higher proliferation markers *Ccnd1* (cyclin D1) and *Cdk4*. In human UACC-3199 cells, low BRCA-1 was paralleled by constitutive high AhR expression; the treatment with αNF rescued BRCA-1 and ERα, while enhancing preferential expression of *CYP1A1* compared to *CYP1B1*. Conversely, in MCF-7 cells, αNF antagonized estradiol-dependent activation of BRCA-1 without effects on expression of ERα. TNBC exhibited increased basal *AhR* and *BRCA-1* promoter CpG methylation compared to LUM-A, LUM-B, and HER-2-positive breast tumors.

**Conclusions:**

Constitutive AhR expression coupled to *BRCA-1* promoter CpG hypermethylation may be predictive markers of ERα-negative breast tumor development. Regimens based on selected AhR modulators (SAhRMs) may be useful for therapy against ERα-negative tumors, and possibly, TNBC with increased AhR and hypermethylated *BRCA-1* gene.

## Background

Germline mutations in the *BRCA-1* gene confer a high probability of developing breast (~65 %) and ovarian (~40 %) tumors [1–6]. Breast tumors lacking BRCA-1 tend to be triple-negative (TNBC) basal-like characterized by reduced expression of estrogen receptor-α (ERα), progesterone receptor (PR), and epidermal growth factor receptor-2 (HER-2) [7]. However, in spite of the high penetrance, *BRCA-1* mutations explain only a small percentage (5-10 %) of breast tumor cases [8]. Sporadic breast tumors do not harbor somatic mutations in *BRCA-1* but express low or undetectable BRCA-1 [9–13].

A mechanism that may contribute to reducing expression of *BRCA-1* in sporadic breast cancers is epigenetic inactivation [14], which refers to modifications in DNA CpG methylation, histone posttranslational modifications, chromatin remodeling factors, and non-coding RNAs [15]. Various degrees of *BRCA-1* promoter CpG methylation have been observed in sporadic breast tumors [16] ranging from ~10 to 85 % depending on tumor type (ductal invasive > lobulo-alveolar) [17–23]. Causes contributing to *BRCA-1* silencing remain largely unknown. Sporadic breast tumors tend to display characteristics of *BRCA-1* mutation cancers (i.e. BRCAness) [24]. These include a high degree of correlation (~75 %) between hypermethylation of the *BRCA-1* and ERα (*ESR1*) genes, and reduced expression of BRCA-1 and ERα [25–29]. Therefore, unraveling the cellular processes that place CpG methylation marks on the *BRCA-1* gene [30] may assist with the formulation of therapies against loss of BRCA-1 expression in *BRCA-1* mutation carriers [31] and non-*BRCA-1* mutation patients [32].

Agonists of the aromatic hydrocarbon receptor (AhR) are ubiquitous in the environment and include dietary compounds, metabolites of fatty acids, industrial xenobiotics, and skin photoproducts generated through exposure to ultraviolet radiation [33]. Importantly, the expression of the AhR and downstream gene targets such as *CYP1B1* are increased in human and rodent mammary tumors [34, 35]. Consequently, the use of selective modulators of the AhR (SAhRMs) has been proposed in breast cancer therapy [36].

Previously, we reported that AhR agonists repressed estradiol (E2)-dependent *BRCA-1* transcription in human breast cancer cells [37–41]. This repressive effect was linked to increased recruitment to the *BRCA-1* promoter of the activated AhR and other factors associated with the epigenetic machinery [42] including DNA methyl-transferase-1, (DNMT-1), DNMT-3a and -3b; methyl-binding domain protein-2 (MBD2); and placement of histone-3 trimethylation marks on lysine-9 (H3K9me3) [43]. In AhR-activated human breast cancer cells, the pattern of *BRCA-1* promoter CpG methylation [44] coincided with the one detected in human sporadic breast tumors [45, 46]. Recently, using a rodent model we found that gestational activation of the AhR increased CpG methylation of the *Brca-1* gene while reducing BRCA-1 expression in mammary tissue of female offspring. The latter changes were overridden by gestational pretreatment with an AhR antagonist [47]. These cumulative data draw attention to the fact alterations of AhR expression and activity may play a role in the etiology of breast tumorigenesis. Nevertheless, the connection between higher AhR expression and/or activation and *BRCA-1* promoter hypermethylation in breast tumors has not been investigated.

This study reports that rat mammary tumors induced with the AhR-agonist 7,12-dimethyl-benzo(a)anthracene (DMBA) [48] had augmented CpG methylation of the *Brca-1* gene; higher expression of *Ahr*, *Cyp1b*, and proliferation markers (*Cdk4*, *Ccnd1*); and diminished expression of BRCA-1 and ERα. In cell culture experiments, the treatment with α-naphthoflavone (αNF), a prototype SAhRM, exerted cell line-specific effects: in ERα-negative human UACC-3199 sporadic breast cancer cell line, it rescued BRCA-1 and ERα expression, while inducing *CYP1A1*; in ERα-positive MCF-7 breast cancer cells, αNF antagonized E2-dependent stimulation of BRCA-1 without affecting ERα expression. Finally, we document that human TNBC had higher *AhR* expression and *BRCA-1* promoter CpG methylation compared to human luminal-A (LUM-A), LUM-B, and HER-2-positive breast tumors. We conclude that constitutive high expression of *AhR* associated with *BRCA-1* gene hypermethylation may be prognostic markers of ERα-negative breast tumor development. Therapies based on SAhRMs may hold promise for rescue of BRCA-1 and ERα expression in ERα-negative breast cancers.

## Methods

### Animal experiments

Weaned female Sprague–Dawley rats and AIN-76A diet were purchased from Harlan Laboratories (Houston, Texas). At day 50 of age, 8 animals/group (*n* = 8) were assigned to either a sesame oil vehicle control group, or a treatment group receiving 10 mg/animal of DMBA (Sigma-Aldrich, St. Louis, MO) by oral gavage [48]. Animals were palpated weekly, and mammary tumors were collected when they reached a diameter of 1 cm. Animals were sacrificed according to a protocol approved by the IACUC Committee of the University of Arizona. Mammary gland tissues and tumors were collected and stored frozen until further analysis.

### Cell culture experiments

Human MCF-7 and UACC-3199 breast cancer cells were obtained from the American Type Culture Collection (Manassas, VA). MCF-7 and UACC-3199 cells were maintained, respectively, in Dulbecco’s Modified Eagles Media (DMEM) or RPMI 1640 media (Mediatech, Manassas, VA) supplemented with 10 % fetal calf serum (FCS) (Hyclone Laboratories, Logan, UT). αNF and E2 for cell culture experiments were obtained from Sigma-Aldrich (St. Louis, MO). For experiments with αNF and E2, cells were plated in 6-well plates at a density of 5 × 10^5^/cells/well. Then, after 24 h cells were cultured for an additional 72 h in phenol-red free DMEM (MCF-7) or RPMI (UACC-3199) supplemented with 10 % charcoal-stripped FCS plus 2 μM αNF in the presence or absence of 10 nM E2 [42]. For Western blotting, at the end of the incubation period, cells were washed with ice-cold phosphate buffer saline (PBS) and scraped with cold lysis buffer containing protease inhibitor. For mRNA studies, at the end of the incubation period, cells were washed with ice-cold PBS. Extraction of RNA was carried out using Triazol Reagent (ThermoFisher Scientific, Grand Island, NY). Cell extracts and RNA samples were stored frozen at -20 °C until further use.

### Breast tumor collection

Human normal and breast tumor tissue sections were obtained de-identified from the University of Arizona Cancer Center Tissue Acquisition and Cellular/Molecular Analysis Shared Resource with the approval from the Institutional Review Board of the University of Arizona, Approval Form#F309. No patient-level correlations between gene activation information and individual patient-data were performed, according to U.S. Department of Human Health Services and Federal Drug Administration regulations, and in compliance with the World Medical Association Declaration of Helsinki (http://www.wma.net/en/30publications/10policies/b3/index.html). The presence of tumor in each sample was confirmed by a staff pathologist and classified according to the following criteria: (i) TNBC: basal-like, and cytokeratin-, ERα-, PR-, HER-2-, and epidermal growth factor-negative; (ii) HER-2-positive: HER-2-positive and ERα-negative; (iii) LUM-A: ERα-positive and/or PR-positive, and HER-2-negative; and (iv) LUM-B: ERα-positive and/or PR-positive, and HER-2-positive. As controls, we also obtained sections of non-tumor tissue from the region surrounding TNBC and LUM-B tumors.

### Western blot analyses

Western blot analyses were performed as previously described [47]. Immunoblotting was carried out with antibodies against human BRCA-1 (Cat. #9010); glyceraldehyde 3-phosphate dehydrogenase (GAPDH) (Cat. #2118) (Cell Signaling Technology, Beverly, MA); rat BRCA-1 (Cat. #sc-642); AhR (Cat. #sc-5579); and ERα (Cat. #sc-542) (Santa Cruz Biotechnology, Dallas, TX). Immunocomplexes were detected using enhanced chemiluminescence (GE Healthcare Life Sciences, Little Chalfont, UK). The GAPDH protein was used as an internal control for normalization of protein expression.

### Promoter CpG methylation

Measurements of rat *Brca-1* promoter CpG methylation were carried out as described previously [47]. Briefly, genomic DNA was isolated from ~30 mg of mammary tissue using the DNeasy Blood & Tissue Kit (Qiagen, Valencia, CA). Then, DNA (1 μg) was subjected to bisulfite modification using the CpGenome DNA Modification Kit (Millipore, Billerica, MA). In preliminary experiments, we verified that the number of cycles for semiquantitative amplification of the rat *Brca-1* promoter fragment with unmethylated (U)- and methylated (M)-specific primers was performed in the linear range (Fig. 2a). Then, the bisulfite-modified genomic DNA obtained from 8 animals/group (*n* = 8) was analyzed by PCR as follows: 1 cycle at 95 °C for 5 min; 37 cycles at 95 °C for 45 s, 55 °C (U) and 59 °C (M) for 45 s, and 72 °C for 1 min; and 1 cycle at 72 °C for 5 min. Briefly, reactions were carried out at a final volume of 25 μL consisting of the following master mix: bisulfite-modified DNA, JumpStart Taq DNA polymerase, 1X PCR buffer, 2.0 mM MgCl_2_, 200 mM dNTPs, 1 μL each of forward and reverse primers. The PCR amplification products were separated on 2 % agarose gels and visualized using ethidium bromide staining. The rat *Brca-1* amplicon was of the expected size (142 bp) and its authenticity to the rat *Brca-1* gene [49] was confirmed by direct sequencing. The rat *Brca-1* primers synthesized by Sigma-Aldrich (St. Louis, MO) were: U-sense: 5’-GTGAGAAGGTTTTTGTTGTATT-3’, and U-antisense: 5’-CCAATTCCAACATACATTACA-3’; M-sense: 5’-GCGAGAAGGTTTTTGTTGTATC-3’, and M-antisense: 5’-ACCAATTCCAACATACATTACG-3’.

Quantitative (qPCR) analysis of human *BRCA-1* promoter CpG methylation in control breast tissue and breast tumors was performed in bisulfonated genomic DNA using the following primers synthesized by Sigma-Aldrich (St. Louis, MO): U-sense: 5'-TTGGTTTTTGTGGTAATGGAAAAGTGT-3', and U-antisense: 5'-CAAAAAATCTCAACAAACTCACACCA-3’; M-sense: 5’-TGGTAACGGAAAAGCG-3’, and M-antisense 5’**-**ATCTCAACGAACTCACGC**-**3’. The qPCR was carried out in a volume of 10 μL consisting of the following master mix: 5 μL of SYBER Green mix (Life Technologies, Grand Island, NY), 1 μL each of forward and reverse primers, 2 μL nuclease-free water, and 1 μL of bisulfonated genomic DNA.

### mRNA analyses

Sections of normal mammary gland and mammary tumor tissues from 8 animals/group (*n* = 8) were homogenized (1 mL/40 mg of tissue) of QIAzol Reagent (Invitrogen, Carlsbad, CA). Total RNA was purified using RNeasy Lipid Tissue Mini Kit as per manufacturer’s instructions (Qiagen, Valencia, CA) [47]. Concentrations and quality of RNA were verified using the Nanodrop1000 Spectrophotometer (Thermo Scientific, Wilmington, DE). Equal amounts of total RNA (500 ng) were transcribed into cDNA using ISCRIPT supermix kit (Bio-Rad Laboratories, Hercules, CA). Next, cDNA aliquots were analyzed by qPCR using the SYBR Green PCR Reagents kit (Life Technologies, Grand Island, NY). Briefly, reactions were run at a final volume of 25 μL consisting of the following master mix: 12.5 μL of SYBR Green mix, 1 μL each of forward and reverse primers, 9.5 μL nuclease-free water, and 1 μL cDNA. Amplification of *Gapdh* mRNA was used for normalization of transcript levels. The rat primer (Sigma-Aldrich, St. Louis, MO) sequences were:

*Ahr*, sense: 5’-CTGGCAATGAATTTCCAAGGGAG-3’; 5’; antisense: CTTTCTCCAGTCTTAATCATGCG-3’; *Cyp1a1*, sense: 5’-GCCTTCACATCAGCCACAGA-3’, antisense: 5’-TTGTGACTCTAACCACCCAGAATC-3’; *Cyp1b1*, sense: 5’-TCAACCGCAACTTCAGCAACTTC-3’; antisense: 5-AGGTGTTGGCAGTGGTGGCAT-3’; *Cdk4*, sense: 5’-TGCAACGCCTGTGGATATGT-3’, antisense: 5’-CAGATTCCTCCATCTCCGGC-3’; *Ccnd1* (cyclin D1), sense: 5’-CTGGCCATGAACTACCTGGA-3’, antisense: 5’-GTCACACTTGATCACTCTGG-3’; *Gapdh*, sense: 5’-TGGTGAAGGTCGGTGTGAAC-3’; antisense: 5’-AGGGGTCGTTGATGGCAACA-3’. For cell culture experiments with human UACC-3199 breast cancer cells, the primer (Sigma-Aldrich, St. Louis, MO) sequences were: *BRCA-1*, sense: 5′-AGCTCGCTGAGACTTCCTGGA-3′, antisense: 5′-CAATTCAATGTAGACAGACGT-3′; *GAPDH*, sense: 5’-ACCCACTCCTCCACCTTT-3’, antisense: 5’-CTCTTGTGCTCTTGCTGGG-3’; *CYP1A1*, sense: 5’-TAACATCGTCTTGGACCTCTTTG-3’, antisense: 5’-GTCGATAGCACCATCAGGGGT-3’; *CYP1B1*, sense: 5’-AACGTCATGAGTGCCGTGTGT-3’, antisense: 5’-GGCCGGTACGTTCTCCAAATC-3’. For *AhR* measurements in human breast tissues and tumors, primer sequences (Sigma Aldrich, St. Louis, MO) were: sense: 5’-GAAGCCGGTGCAGAAAACAG-3’, antisense: 5’-GCCGCTTGGAAGGATTTGAC-3’.

### Statistical methods

Densitometry after Western blotting and CpG methylation analyses were performed using Kodak ID Image Analysis Software (Eastman Kodak Company, Rochester, NY). Statistical analyses were performed using Prism 5.0 (GraphPad Software Inc., La Jolla, CA) [47]. Data were analyzed by 1-way ANOVA. Post-hoc multiple comparisons among all means were conducted using Tukey’s Test after main effects and interactions were found to be significant at *P* ≤ 0.05. Data were presented as means ± SEM and statistical differences highlighted with different letters or asterisks.

## Results

### BRCA-1 expression in mammary tumors

**Fig. 1:**
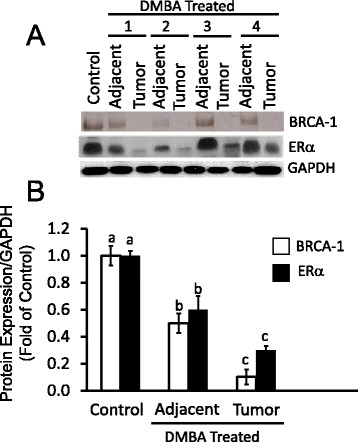
Expression of BRCA-1 and ERα are reduced in DMBA-induced mammary adjacent tissues and tumors. **a** Bands are representative immunocomplexes for BRCA-1 and ERα in control mammary gland, and mammary adjacent tissue and tumors obtained from four (1 through 4) DMBA-treated rats; **b** Bars represent means ± SEM of quantitation (fold change of control) of BRCA-1 and ERα protein corrected for GAPDH protein as internal standard in mammary adjacent tissues and tumors from 8 animals/group (*n* = 8). Different letters represent statistical differences (*P* < 0.05)

Previous studies documented a high degree (~75 %) of correlation between loss of *BRCA-1* and reduced ERα expression in human breast tumors [27, 28]. Results of BRCA-1 and ERα protein expression in control mammary tissue, and in adjacent normal mammary tissues and tumors obtained from animals treated with DMBA are presented in Fig. 1. Compared to control mammary gland, BRCA-1 expression (Fig. 1a) was reduced by an average 50 % in peritumoral mammary tissue (Fig. 1b). BRCA-1 protein levels were reduced by an additional ~40 % in DMBA-induced mammary tumors. Similarly, we found that compared to control mammary tissue, ERα levels were reduced by an average 40 % and 70 % respectively, in DMBA-treated but apparently normal mammary gland, and mammary tumors.

### *Brca-1* promoter CpG methylation in mammary tumors

**Fig. 2:**
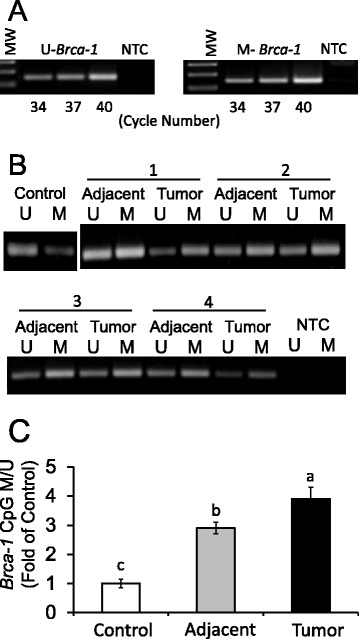
*Brca-1* promoter methylation is increased in DMBA-induced rat mammary adjacent tissues and tumors. **a** Cycle number and no-template control (NTC) for amplification of rat *Brca-1* promoter with U- and M-specific primers. MW, molecular weight markers; **b** Methylation status of *Brca-1* promoter in control mammary gland, and in adjacent mammary tissues and tumors of four representative (1-4) animals; C) Quantitation from genomic DNA of *Brca-1* promoter methylation status (M/U ratio) compared to control from 8 animals/group (*n* = 8). Means ± SEM without a common letter differ (*P* < 0.05)

To examine if the reduction in BRCA-1 expression in DMBA-treated animals was related to changes in *Brca-1* promoter CpG methylation status, we extracted genomic DNA from control mammary gland, and adjacent mammary tissues and tumors from DMBA-treated animals. In control experiments, we ascertained that rat bisulfonated genomic DNA obtained from control mammary tissue was amplified in the linear range with U- and M-specific *Brca-1* oligonucleotides, and *Brca*-1 amplicons were of the expected size (142 bp) (Fig. 2a). Turning to changes in *Brca-1* promoter CpG methylation (Fig. 2b), we found that compared to control, the adjacent mammary gland isolated from DMBA-treated animals had an average 1.9-fold increase in *Brca-1* promoter CpG methylation (Fig. 2c), which was increased on average an additional ~1.0-fold in DMBA-induced tumors (Fig. 2c). These data suggested that the *Brca-1* gene was a target for repression via CpG methylation in mammary tissue of animals treated with the AhR agonist and mammary carcinogen, DMBA.

### *AhR* expression and activation in mammary tumors

**Fig. 3:**
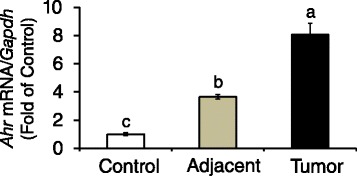
Expression of *Ahr* is increased in DMBA-induced rat mammary adjacent tissues and tumors. Bars represent means ± SEM of quantitation (fold change of control) of *Ahr* mRNA corrected for *Gapdh* mRNA as internal standard in control and DMBA-induced adjacent mammary tissues and tumors from 8 animals/group (*n* = 8). Different letters represent statistical differences (*P* < 0.05)

**Fig. 4:**
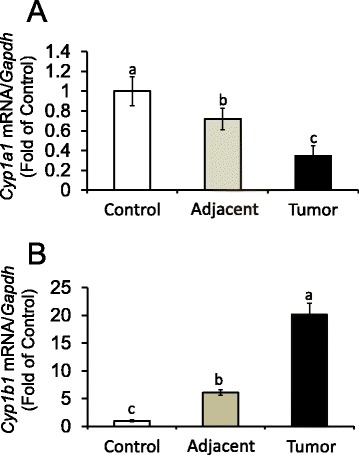
Differential regulation of *Cyp1a1* and *Cyp1b1* in DMBA-induced rat mammary adjacent tissues and tumors. Bars represent means ± SEM of quantitation (fold change of control) of (**a**) *Cyp1a1* and (**b**) *Cyp1b1* mRNA corrected for *Gapdh* mRNA as internal standard in control and DMBA-induced adjacent mammary tissues and tumors from 8 animals/group (*n* = 8). Different letters represent statistical differences (*P* < 0.05)

Focusing on measurements of *Ahr* expression and activation, we first examined changes in *Ahr* in mammary tissue of control animals, and peritumoral and tumor tissues obtained from DMBA treated animals (Fig. 3). Compared to control, levels of *Ahr* were increased ~2.7-fold in peritumoral tissues; *Ahr* expression was increased an additional ~4.5-fold in DMBA-induced mammary tumors. In human breast cancer cell lines, higher *CYP1B1* expression over *CYP1A1* has been related to higher AhR expression and ERα-negative status [50]. Therefore, we measured changes in expression of *Cyp1a1* and C*yp1b1* as controls for AhR pathway activation. Basal *Cyp1a1* was reduced by 30 and 70 % respectively in adjacent mammary gland and mammary tumors (Fig. 4a). Conversely, *Cyp1b1* levels were markedly increased, on average ~5.0 and 14.0-fold of control, respectively in peritumoral tissue and mammary tumors (Fig. 4b). These data indicated that constitutive overexpression of the *Ahr* in rat mammary tumors was coupled with differential regulation on the *Cyp1a1* (repression) and *Cyp1b1* (activation) target genes.

### Proliferation markers in mammary tumors

**Fig. 5:**
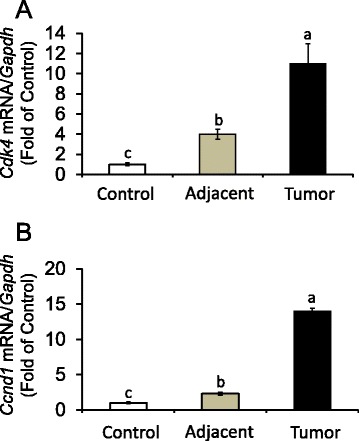
Expression of *Cdk4* and *Ccnd1* (cyclin D1) are increased in DMBA-induced rat mammary adjacent tissues and tumors. Bars represent means ± SEM of quantitation (fold change of control) of (**a**) *Cdk4* and (**b**) *Ccnd1*(cyclin D1) mRNA corrected for *Gapdh* mRNA as internal standard in control and DMBA-induced adjacent mammary tissues and tumors from 8 animals/group (*n* = 8). Different letters represent statistical differences (*P* < 0.05)

Previous investigations documented that increased expression and activation of the AhR may be associated with mitogenic responses [51, 52], and increased *Cdk4* levels in rat [47] and human [53] mammary cells. Based on this information, we compared *Ccnd1* (cyclin D1) and *Cdk4* expression in control mammary gland, and adjacent mammary tissues and tumors obtained from animals treated with DMBA (Fig. 5). We noticed that compared to control, in DMBA-treated animals levels of *Cdk4* (Fig. 5a) and *Ccnd1* (Fig. 5b) were increased respectively an average ~3.0- and 1.0-fold in adjacent mammary tissues; and an additional ~6.0- and 12.0-fold increase was seen, respectively, for *Cdk4* and *Ccnd1*, in mammary tumors. Taken together, animal results suggested that constitutive high *Ahr* expression and pathway activation on the *Cyp1b1* gene were linked to induction of mammary tumorigenesis associated with reduced expression of BRCA-1 and ERα.

### Targeting of AhR with αNF in human breast cancer cells

**Fig. 6:**
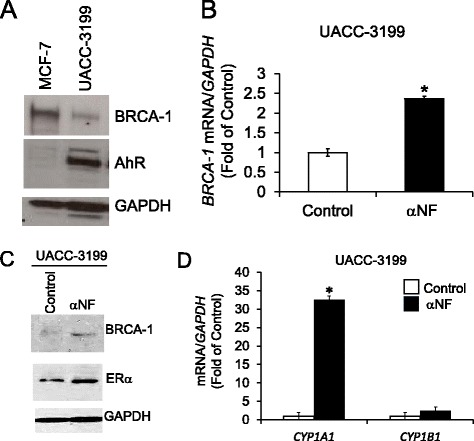
Rescue of BRCA-1 and ERα expression in sporadic UACC-3199 breast cancer cells with αNF. **a** Bands are representative baseline immunocomplexes detected by Western blotting for BRCA-1 and AhR protein expression in MCF-7 and UACC-3199 breast cancer cells cultured respectively in control phenol-red free DMEM or RPMI 1640 media supplemented with 10 % charcoal-stripped FCS; **b** UACC-3199 breast cancer cells were cultured in control phenol-red free RPMI plus 10 % charcoal-stripped FCS in the absence (control) or presence of αNF (2 μM for 72 h). Bars represent means ± SEM of quantitation of mRNA (fold change of control) performed twice in duplicate (n = 4) with four repeated measures/sample. *BRCA-1* mRNA was corrected for *GAPDH* mRNA as internal standard; **c** Bands are immunocomplexes detected by Western blotting for BRCA-1 and ERα in UACC-3199 breast cancer cells cultured in phenol-red free RPMI plus 10 % charcoal-stripped FCS in the absence (control) or presence of αNF (2 μM for 72 h). GAPDH bands are internal standards for Western blotting; **d** Bars represent means ± SEM of quantitation of *CYP1A1* and *CYP1B1* mRNA (fold change of control) performed twice in duplicate (*n* = 4) with four repeated measures/sample, and corrected for *GAPDH* mRNA as internal standard. In (**b**) and (**d**) Asterisks represent statistical differences (*P* < 0.05) compared to control

Increased expression and activation of the AhR may contribute to epigenetic remodeling during early breast carcinogenesis [54], whereas loss of BRCA-1 associates with ERα-negativity in hereditary and sporadic breast tumors [27]. Therefore, we compared the expression of BRCA-1 and AhR in human ERα-positive MCF-7, and ERα-negative UACC-3199 sporadic, breast cancer cells. We selected these cell lines because MCF-7 cells express wild-type *BRCA-1* and are ERα-positive. Conversely, UACC-3199 cells have wild-type but hypermethylated, *BRCA-1* [21, 55], and express low levels of ERα [56]. Results of Western blots informed that expression of BRCA-1 was ~5.0-fold higher in MCF-7 compared to UACC-3199 cells (Fig. 6a). Conversely, the expression of the AhR was notably higher (~15.0-fold) in UACC-3199 compared to MCF-7 cells.

**Fig. 7:**
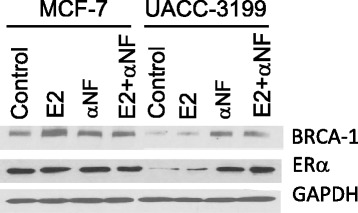
Differential effects of αNF on BRCA-1 and ERα expression in MCF-7 and UACC-3199 breast cancer cells. Cells were cultured for 72 h in control phenol red-free media (DMEM for MCF-7; RPMI for UACC-31299) supplemented with 10 % charcoal-stripped FCS in the presence or absence of 10 nM E2, alone or in combination with 2 μM αNF. Bands are representative immunocomplexes detected by Western blotting for BRCA-1, ERα, and GAPDH from two independent experiments performed in duplicate (*n* = 4)

In previous studies with MCF-7 cells, we used αNF to reverse the repressive effects of AhR agonists on BRCA-1 expression [38]. We then extended these studies to UACC-3199 breast cancer cells. Results depicted in Fig. 6b revealed that the treatment with αNF increased (~1.4-fold of control) *BRCA-1* mRNA; this change was associated with a ~2.0-fold upregulation of BRCA-1 and ERα expression (Fig. 6c). Turning to other biological changes that occurred in UACC-3199 cells along with reactivation of BRCA-1 by αNF, we detected a large increase (~32-fold of control) in *CYP1A1* expression with only modest effects (~1.5-fold increase compared to control) on *CYP1B1* (Fig. 6d). Then, we compared the effects of αNF on BRCA-1 and ERα expression in MCF-7 and UACC-3199 breast cancer cells. Results illustrated in Fig. 7 confirmed that αNF increased ~2.0- and 3.0-fold of control respectively, BRCA-1 and ERα in UACC-3199 cells, which were however refractory to the treatment with E2 alone or in combination with αNF. On the other hand, as previously reported by our group [41, 42], the treatment of MCF-7 cells with αNF antagonized the E2-dependent induction of BRCA-1. Overall, these cell culture studies implied that the effects of αNF, selected as a prototype AhR antagonist, were influenced by cell-context and ERα status, i.e. αNF rescued BRCA-1 and ERα expression in sporadic and ERα-negative UACC-3199 breast cancer cells carrying hypermethylated *BRCA-1*. Conversely, αNF antagonized E2-dependent stimulation of BRCA-1 expression in ERα-positive MCF-7 breast cancer cells.

### BRCA-1 promoter methylation and AhR expression in human breast tumors

**Fig. 8:**
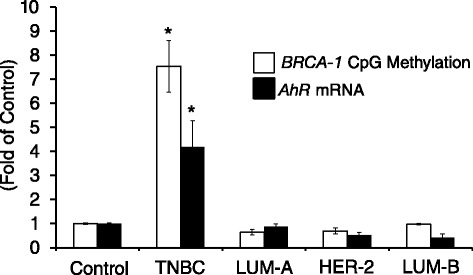
Human TNBC harbor constitutive *AhR* expression and increased *BRCA-1* promoter CpG methylation. Bars represent quantitation of *BRCA-1* promoter CpG methylation (M/U ratio) and *AhR* expression in human TNBC (*n* = 4), LUM-A (*n* = 5), LUM-B (*n* = 4), and HER-2-positive (*n* = 5) breast tumors. Asterisks represent statistical differences (*P* < 0.05) compared to non-tumor breast tissue control

Next, we wished to explore if differential expression of *AhR* and *BRCA-1* promoter CpG methylation associated with pathological classification of human breast tumor subtypes based on receptor status. Therefore, we compared the level of *BRCA-1* promoter CpG methylation in genomic DNA obtained from control breast tissue and various breast tumor subtypes including TNBC, LUM-A, HER-2-positive, and LUM-B. On average, we observed that *BRCA-1* promoter methylation (M/U ratio) was increased ~6.6-fold in TBNC compared to non-tumor breast tissue (Fig. 8). Conversely, compared to non-tumor tissue, there were no differences in the amount of *BRCA-1* promoter methylation in LUM-A, LUM-B, and HER-2-positive breast tumors. Interestingly, the increased *BRCA-1* promoter methylation in TNBC correlated with increased expression (~3.0-fold of control) of *AhR*. Overall, these results denoted that coordinated increase in *AhR* expression and *BRCA-1* gene hypermethylation may be molecular markers of TNBC.

## Discussion

Earlier studies documented that the AhR is overexpressed and constitutively activate in rodent and human mammary tumors [35]. These findings attributed to environmental and endogenous factors that activate the AhR a role in breast tumorigenesis. Our prior cell culture [37–44] and rodent [47] model investigations of breast cancer provided evidence that the *BRCA-1* gene was a molecular target for the AhR and various chromatin remodeling factors. Specifically, the recruitment of the activated AhR, DNMTs, and MBD-2 to the *BRCA-1* gene culminated with placement of repressive histone (H3K9me3) and DNA (CpG methylation) marks, and downregulation of BRCA-1 expression.

The first objective of this study was to investigate the association between AhR expression and/or activation and *Brca-1* promoter methylation status in mammary tumors. For this purpose, we adopted the DMBA-rat mammary tumor model based on the knowledge DMBA is a strong AhR agonist [33] and mammary carcinogen [48, 57]. The upregulation of *Ahr* and *Cyp1b1* were paralleled by increased *Brca-1* CpG methylation, and reduced expression of BRCA-1 and ERα in mammary tumors induced with DMBA. Also, the reduction in BRCA-1 expression observed in peritumoral tissue suggested that *Brca-1* CpG methylation may be an epigenetic event that occurs prior to overt mammary tumor formation linked to *Ahr* overexpression and/or activation. This interpretation may have prognostic value since adjacent non-tumor mammary tissue from DMBA-treated animals had also increased expression of the proliferation markers *Cdk4* and *Ccnd1* (cyclin D1). Overall, results of animal experiments linked higher *AhR* expression and activity on the *Cyp1b1* gene to increased risk of mammary tumorigenesis [34, 48, 54, 57, 58] via epigenetic silencing of *Brca-1*.

The reduction in ERα expression observed in adjacent mammary gland and mammary tumors of DMBA-treated animals was consistent with previous reports of reduced ERα in familial BRCA-1 tumors [25, 26], and sporadic breast cancers with hypermethylated *BRCA-1* [28]. The ERα and the BRCA-1 participate in a positive feed-back loop whereby the ERα upregulates *BRCA-1* [38], which in turn stimulates ERα expression [27]. Therefore, AhR-dependent repression of *BRCA-1* via increased CpG methylation may disrupt this positive feedback loop between *BRCA-1* and *ER*α and favor the development of ERα- and BRCA-1-negative breast tumors.

Turning to markers of AhR activation, we measured increased *Cyp1b1* in adjacent mammary gland and mammary tumors of DMBA-treated animals. This accumulation was consistent with previous studies reporting stimulation of *Cyp1b1* in rat models of mammary tumorigenesis [34, 48]. The CYP1B1 enzyme catalyzes the production from E2 of mutagenic 4-hydroxy-E2 (4OH-E2) [59, 60]. It is feasible that the constitutive activation of the AhR/CYP1B1 axis may have the synergistic effect of increasing DNA damage via increased production of mutagenic 4OH-E2 while impairing DNA repair functions controlled by BRCA-1. Conversely, we found that *Cyp1a1* was reduced in adjacent mammary gland and mammary tumors of DMBA-treated animals. Consistent with these findings, earlier studies documented preferential repression of *Cyp1a1* in DMBA-induced mammary tumors [48], as well as in human invasive ductal carcinomas [61, 62] and breast cancer cells lacking the ERα [50, 63]. Furthermore, reduced CYP1A1 enzymatic activity has been linked to constitutive activation of the AhR [64] and resistance of breast cancer cells to apoptosis induced by DMBA [65].

To further elucidate the cross-talk between expression and/or activation of AhR, and *BRCA-1* regulation, we turned to cell culture experiments using UACC-3199 sporadic breast cancer cells, which possess hypermethylated BRCA-1 promoter [21, 55] and express low ERα [56]. Compared to MCF-7 cells, UACC-3199 cells had higher basal AhR, but lower BRCA-1. Therefore, we tested whether or not treatment of UACC-3199 cells with the AhR antagonist αNF rescued BRCA-1 expression. The rationale for this approach was based on our previous studies showing that BRCA-1 silencing by AhR agonists was reversed by cotreatment with α-NF [38]. The mechanisms of action of αNF as an AhR antagonist and anticarcinogen have been related respectively, to reduction of transcriptionally active nuclear AhR complexes [66, 67], and inhibition of 4OH-E2 production by CYP1B1 [68]. The rescue of BRCA-1 and ERα by αNF in UACC-3199 breast cancer cells were biological changes associated with preferential induction of *CYP1A1*. Conversely, αNF did not affect ERα levels, but antagonized E2-dependent activation of BRCA expression, in ERα-positive MCF-7 cells. The latter findings were in accord with our previous reports documenting repression by αNF and 3-methoxy-4-naphthoflavone, another antagonist of the AhR, of E2-dependent transcriptional activation of the *BRCA-1* gene [42]. These differential effects of αNF on BRCA-1 and ERα expression could be attributed to interactions between agonist/antagonist activities on the AhR and ERα status [69]. This AhR-ERα cross-talk could be exploited for the development of strategies aimed at the reactivation of BRCA-1 and ERα in ERα-negative and AhR-overexpressing tumors.

We further extended our studies of BRCA-1/AhR cross-talk to human breast tumors, and found that compared to LUM-A, LUM-B, and HER-2-positive tumors, TNBC had higher *AhR* and *BRCA-1* CpG methylation. These observations provided additional support to the hypothesis that constitutive *AhR* expression may be associated with hypermethylation of the *BRCA-1* promoter and the development of TNBC. It remains unknown whether the reduced ERα expression in DMBA-induced tumors, UACC-3199 cells, and TNBC tumors may be due to hypermethylation, or disruption of expression of transcription factors that regulate transcription, of the ERα (*ERS1)* gene. Answering these queries may assist with the development of strategies for coordinate epigenetic reactivation of *BRCA-1* and *ESR1* in ERα-negative breast tissues.

## Conclusions

Many studies have effectively utilized the AhR-agonist and mammary carcinogen DMBA to examine the molecular pathways that contribute to breast cancer and efficacy of therapies [57]. To our knowledge, this is the first study linking constitutive overexpression of the *AhR* to *BRCA-1* promoter hypermethylation in DMBA-induced mammary tumors and human TNBC. The potential prognostic significance of the current findings is underscored by the fact the AhR is constitutively active in ERα-negative human breast tumor cells [34, 35, 50, 61]. Ongoing studies in our laboratory are using in vitro and vivo models to explore the effects of AhR knockout on epigenetic regulation of *BRCA-1* and *ESR1* (ERα), and the preventative effects of AhR antagonists. Progress in these areas may help clarifying a causative role for the AhR in breast tumorigenesis and assist with the development of risk models for *BRCA-1* mutation carriers [70, 71] and sporadic TNBC, for which therapy options remains an intensive area of investigation [72, 73].

## Abbreviations

4OH-E2: 4-hydroxy-estradiol
AhR: Aromatic hydrocarbon receptor
αNF: α-naphthoflavone
DMBA: 7,12-dimethyl-benzo(a)anthracene
DMEM: Dulbecco’s Modified Eagles Media
DNMT: DNA methyl-transferase
E2: Estradiol
ERα: Estrogen receptor-α
FCS: Fetal calf serum
GAPDH: Glyceraldehyde 3-phosphate dehydrogenase
HER-2: Epidermal growth factor receptor-2
H3K9me3: Histone-3 trimethylated at lysine-9
M: LOH: Loss of heterozygosity
LUM-A: Luminal-A
LUM-B: Luminal-B
MBD2: Methyl-binding domain protein-2
M: Methylated specific primers
PBS: Phosphate buffer saline
PR: Progesterone receptor
SAhRMs: Selective modulators of the AhR
TNBC: Triple-negative breast cancer
qPCR: quantitative PCR
U: Unmethylated specific primers
